# Application of CRISPR/Cas9 Gene Editing System on MDV-1 Genome for the Study of Gene Function

**DOI:** 10.3390/v10060279

**Published:** 2018-05-24

**Authors:** Yaoyao Zhang, Na Tang, Yashar Sadigh, Susan Baigent, Zhiqiang Shen, Venugopal Nair, Yongxiu Yao

**Affiliations:** 1 The Pirbright Institute & UK-China Centre of Excellence for Research on Avian Diseases, Pirbright, Ash Road, Guildford, Surrey GU24 0NF, UK; zhangyaoyao3848@126.com (Y.Z.); na.tang@pirbright.ac.uk (N.T.); yashar.sadigh@pirbright.ac.uk (Y.S.); sue.baigent@pirbright.ac.uk (S.B.); 2 Binzhou Animal Science and Veterinary Medicine Academy & UK-China Centre of Excellence for Research on Avian Diseases, Binzhou 256600, Shandong, China; bzshenzq@163.com

**Keywords:** CRISPR/Cas9, CVI988, *Meq*, *pp38*, deletion

## Abstract

Marek’s disease virus (MDV) is a member of alphaherpesviruses associated with Marek’s disease, a highly contagious neoplastic disease in chickens. Complete sequencing of the viral genome and recombineering techniques using infectious bacterial artificial chromosome (BAC) clones of Marek’s disease virus genome have identified major genes that are associated with pathogenicity. Recent advances in CRISPR/Cas9-based gene editing have given opportunities for precise editing of the viral genome for identifying pathogenic determinants. Here we describe the application of CRISPR/Cas9 gene editing approaches to delete the *Meq* and *pp38* genes from the CVI988 vaccine strain of MDV. This powerful technology will speed up the MDV gene function studies significantly, leading to a better understanding of the molecular mechanisms of MDV pathogenesis.

## 1. Introduction

Marek’s disease virus (MDV) is a member of the genus *Mardivirus*, sub-family *Alphaherpesvirinae* in the family *Herpesviridae*. MDVs are classified into three closely related, but distinct groups. Serotype 1 viruses (GaHV-2) cause an acute lymphoproliferative disease in chickens, resulting in T-cell lymphomas that metastasize to visceral organs and peripheral nerves. Serotypes 2 (GaHV-3) and 3 viruses (MeHV-1), which were isolated from chickens and turkeys, respectively, are nonpathogenic. Marek’s disease (MD) has been controlled by vaccines that include attenuated serotype 1 (CVI988 or Rispens), serotype 2 (SB-1 or 301B/1) and serotype 3 (Herpesvirus of Turkeys [HVT]) virus strains [1,2,3]. One of the major challenges facing the vaccination strategy is the evolution of viruses towards greater virulence, forcing the need to introduce newer vaccines to keep up with rapidly evolving viruses. An extensive knowledge about MDV gene function is crucial to the understanding of viral pathogenicity, hence the vaccine development.

The development of both cosmid DNA and Bacterial artificial chromosome (BAC) technologies has greatly facilitated the introduction of mutations into viral genomes to study gene functions. Using these technologies, the function of several MDV genes has been investigated. Studies have shown that the genes present in long repeat regions of the MDV genome, including viral telomerase RNA [4,5], viral *IL-8* [6,7,8,9], *Meq* [10,11,12], *pp38* [13,14], and *RLORF4* [15], as well as the large subunit of the ribonucleotide reductase (RR) enzyme in the unique long (UL) region [16], play an important role in pathogenesis. Construction of MDV-1 BAC clone BAC20 mutants carrying deletions of the *UL46*, *UL47*, *UL48*, and *UL49*, alone or in different combinations using the BAC mutagenesis system, has demonstrated that the MDV-1 VP22 protein encoded by *UL49* is indispensable for virus growth, whereas VP11/12, VP13/14, and VP16 are nonessential [17]. Application of BAC technology has opened new avenues in basic herpesvirus research, although there are still a number of herpesviruses whose genomes have not been successfully cloned as BAC. Moreover, cloning of viral genomes as BAC plasmid is time consuming, requiring repeated cell culture passages leading to too much attenuation of the vaccine seed virus stocks; however, the subsequent BAC mutagenesis is fast and efficient.

The CRISPR (clustered regularly interspaced short palindromic repeat)/Cas9 system found in several bacteria and archaea embodies—an adaptive immune mechanism that relies on faithful recognition of specific nucleic acid sequences—is the latest development that allows more simplified and efficient gene editing in many settings [18,19,20,21,22,23,24,25,26]. The CRISPR/Cas9 system has been used successfully to generate knockout cells and animals [27,28]. It has also been used to manipulate genomes of several large DNA viruses, including herpes simplex virus type I, adenovirus, pseudorabies virus, vaccinia virus, Epstein-Barr virus, guinea pig cytomegalovirus, and duck enteritis virus [22,29,30,31,32,33,34,35,36,37]. Having demonstrated that the HVT genome can be efficiently edited using the CRISPR/Cas9 system [38,39], we want to know whether the MDV-1 genome can be manipulated using the same system for gene function studies.

Among the more than 100 genes encoded by the MDV [40,41], Meq protein, which is expressed both in lytic and latent infections undisputedly, is the most important viral gene associated with MD oncogenicity [42,43]. *pp38*, another important gene in MDV pathogenesis, is a unique phosphoprotein and is necessary for cytolytic infection of B cells, maintenance of the transformed state but not for cytolytic infection of the feather follicle epithelium, and horizontal spread of MDV [44,45,46]. In the present report, we developed a rapid and efficient method for generation of CVI988 mutant viruses lacking the *Meq* and *pp38* gene, respectively, using a CRISPR/Cas9 editing system. The mutant viruses were characterized by PCR, immunofluorescence assay (IFA) and growth kinetics. These results show that CRISPR/Cas9-mediated gene editing is a simple and rapid approach to manipulate MDV’s viral genome for better understanding of the molecular mechanism of MDV pathogenesis.

## 2. Materials and Methods

### 2.1. Cell Culture and Viruses

Primary chick embryo fibroblasts (CEF) used in this study were prepared from 10-day old Valo SPF embryos. Cells were cultured in M199 medium (Life Technologies, Paisley, UK), supplemented with 5% fetal bovine serum (FBS, Sigma, Dorset, UK), 100 units/mL of penicillin and streptomycin (Life Technology), 0.25µg/mL Fungizone (Sigma), and 10% tryptose phosphate broth (Sigma). Commercial CVI988 vaccine stocks were obtained from Merial Cryomarex Rispens.

### 2.2. Construction of Guide RNA Constructs

The Zhang Lab CRISPR design algorithm (http://crispr.mit.edu/) was used to design guide RNAs targeting both ends of *pp38* and *Meq* genes of the CVI988 virus. The highest scoring gRNAs with a low probability of off target cleavage events were chosen. Three gRNAs from either ends were selected and cloned into CRISPR/Cas9 vector pX330A-1x2 and pX330S-2, respectively, by introducing synthesized oligo-DNA primers corresponding to the target sequence in *Bbs*I restriction sites. The most efficient gRNA from each end was chosen by High Resolution Melting (HRM) analysis after transfection with an individual gRNA construct and infection with the CVI988 virus, as described previously [38]. The chosen gRNA expression cassette from pX330S-2 was then transferred into pX330A-1x2 via *BsaI* site generating pX330A-2xgRNAs vectors. The oligonucleotides used are listed in Table 1.

### 2.3. Generation of Meq and pp38 Gene Deletion CVI988 Virus

The primary CEF were plated into 6-well plates the day before transfection and transfected with Cas9/gRNA expression plasmid, using Lipofectamine^®^ Reagent (Thermo Fisher Scientific, Basingstoke, UK) according to the manufacturer’s instruction. At 8 h post transfection, the CEF cells were infected with CVI988 at 0.01 pfu/cell. The infected CEF were passed 3 days later and the individual plaques were picked and analyzed further.

### 2.4. Characterization of Gene Knockout CV988 Virus

The CEF cells plated in 6-well plates the previous day were infected with parental CVI988 or the plaque purified mutant virus CVI988-Δ*pp38*/CVI988-Δ*Meq*. The infected cells were harvested at 72 h post infection and lysed in 1× squishing buffer (10 mM Tris-HCl, pH 8, 1 mM EDTA, 25 mM NaCl, and 200 µg/mL Proteinase K) at 65 °C for 30 min. PCR was carried out to identify the correct gene knocked-out viruses using primers outside the targeted sites. The PCR products were purified and sequenced.

### 2.5. Immunofluorescence Assay (IFA)

The expression of *pp38* and *Meq* in the mutant virus infected cell was evaluated by IFA using confocal microscopy. The CEF cells grown on coverslips in 24-well plates were infected with the mutant and parental CVI988 viruses for 48–72 h before harvesting. After being fixed with 4% paraformaldehyde and permeabilized with 0.1% Triton X-100, the cells were stained with mAb BD1 for *pp38* expression, FD7 for *Meq* expression and HB3 for *gB* expression. Cell nuclei were then stained with 4,6-diamidino-2-phenylindole (DAPI). Images were taken using a Leica TCS SP5 confocal laser scanning microscope (Leica Microsystems, Wetzlar, Germany).

### 2.6. The Growth Kinetics of the Gene Knockout Viruses

The in vitro growth kinetics of the gene knockout viruses were compared with the parental virus on CEF by qPCR at various time points. One hundred pfu of each virus was inoculated into fresh CEF in 6-well plates. At 0, 24, 48, 72, 96, 120 and 144 h post infection, infected cells were harvested, and DNA was extracted using the DNeasy 96 Blood & Tissue kit (Qiagen, Manchester, UK) for real-time qPCR to determine the in vitro growth kinetics of the viruses, using the methods described previously [47]. Duplex real-time qPCR was used to detect the HVT *SORF1* gene and the chicken ovotransferrin gene-enabled calculation of HVT genome copies per 10,000 cells, using a dilution series of pHVT BAC3 DNA [47] and p-GEM-T-ovo [48] to produce a standard curve. The HVT genome copies per 10,000 cells were plotted against hours of post-infection for each of the viruses.

### 2.7. qRT-PCR for MDV Transcripts and miRNAs

Relative expression levels of MDV-encoded transcripts *Meq*, *pp38*, *ICP4*, *LAT* and host *GAPDH* were measured by qRT-PCR in comparison to the viral gene *gB* transcript. For this, total RNA (1 μg) extracted from infected cells was treated with DNase I (Promega, Southampton, UK) at 37 °C for 1 h, and reverse transcribed using Superscript III (Thermo Fisher Scientific) as per the manufacturer’s protocols. PCR amplification was carried out in a 20 μL reaction volume with 5 μL of RT reaction (1:5 dilution), 0.1–0.15 μM FAM-labeled MGB probe (Thermo Fisher Scientific), 0.2–0.25 μM forward and reverse primers, and 10 μL of Universal PCR Master Mix. The PCR conditions used were: 95 °C for 10 min, followed by 40 cycles at 95 °C for 15 s and 60 °C for 1 min. The details of the oligonucleotide primers and labeled probes are described previously [49]. All real-time qPCR tests were run in triplicate on the ABI Prism 7500 Sequence Detection System (Thermo Fisher Scientific, Basingstoke, UK).

The expression levels of selected miRNAs were analyzed using the TaqMan MicroRNA Assay System (Life Technologies) using 10 ng total RNA as a template for reverse transcription. Each reverse transcription reaction was performed twice independently, and each reaction was tested by PCR in triplicate. All values were normalized to the expression of the endogenous *let-7a*, and levels were calculated as the fold expression change relative to those from the wild type CVI988 infected cells.

## 3. Results

### 3.1. Construction of pp38 Deletion Mutant CVI988-Δpp38 Using CRISPR/Cas9 System

**Figure 1:**
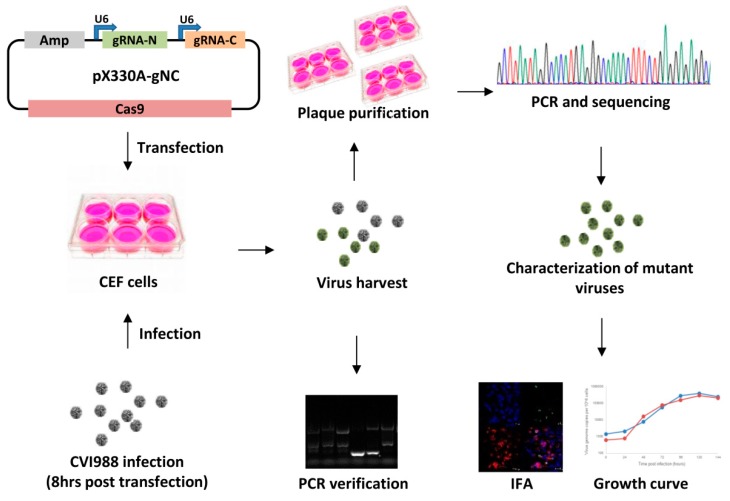
A diagram depicting the protocol used to generate mutant viruses. The gRNA/Cas9 expression plasmid for targeting the gene of interest was transfected into CEF and followed by CVI988 virus infection. The transfected/infected cells were harvested followed by PCR and plaque purification. The purified plaques were then verified by PCR and sequencing. The verified virus was characterized by IFA and growth kinetics studies.

Previously we have demonstrated that HVT genes could be disrupted [38] or the foreign gene could be inserted into the HVT genome efficiently to generate a recombinant HVT vaccine using the CRISPR/Cas9 system [39]. In order to determine whether the CRISPR-Cas9 system could be used to delete genes from viral genomes for gene function studies, we have chosen to knock out *pp38* from CVI988, the vaccine strain of MDV-1. Two gRNAs targeting the 5′ and 3′ end of *pp38* were designed using the CRISPR guide RNA designing software (http://crispr.mit.edu/) and cloned sequentially into the CRISPR/Cas9 vector pX330A-1x2 (Addgene, #1000000055), which co-expressed two gRNAs and Cas9 nuclease to generate a pp38-gNC construct. The primers used are listed in Table 1. The detailed procedure for the generation and characterization of gene knockout viruses is described in Figure 1.

**Figure 2:**
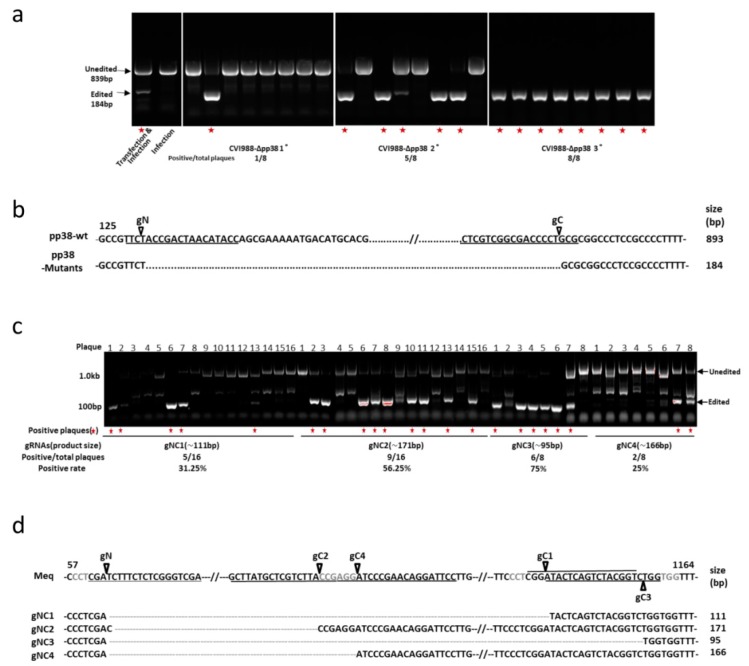
Isolation and characterization of mutant viruses. (**a**) PCR amplification of the edited region, using primers pp38-N-F and pp38-C-R on the cell lysate of transfected/infected cells and plaques after a subsequent three rounds of plaque purification. Red asterisk indicates the plaques containing the edited band. (**b**) The nucleic acid sequences of the truncated/edited PCR product showing the successful deletion of *pp38*. Target sequence is underlined and the cleavage site is indicated by an arrow. (**c**) PCR amplification of the edited region of the *Meq* gene using primers Meq-N-F and Meq-C-R on the cell lysate of plaques with different pairs of guide RNAs, targeting both ends of the *Meq* gene. Red asterisk indicates the plaques containing the edited band. (**d**) The nucleic acid sequences of the truncated/edited PCR product showing the successful deletion of *Meq*. Target sequence is underlined, PAM sequence is in grey and the cleavage site is indicated by an arrow.

Briefly, 8 h post transfection of pp38-gNC into CEF, the cells were infected with the CVI988 virus. The transfected/infected cells were harvested 3 days post infection. 1 × 10^6^ cells were passed by serial dilution until single plaques were observed. 1 × 10^6^ cells were lysed for DNA extraction and subjected to direct PCR using *pp38*-specific primers flanking the region of Cas9 targeting sites. DNA from the virus-infected cells generated an 839 bp PCR product that corresponded to the unedited *pp38* (Figure 2a). In contrast, DNA from the pp38-gNC transfected and virus infected cells, also contained a 184 bp product that corresponded to the deleted region between the two Cas9 cleavage sites in *pp38*. The detection of two bands indicated the presence of a mixed population of both edited and unedited viruses. Virus stocks from eight purified plaques were tested by *pp38* locus-specific PCR to identify any potential mutant viruses. As shown in Figure 2a, one of these stocks contained predominantly the smaller band, indicating the vast majority of these are edited viruses. Following one more round of plaque purification of this plaque, eight plaques were picked and the positive rate of mutant plaques was increasing to 62.5%. Of which, three plaques have the single edited band and two plaques have a mixed population. The purity of the plaque with the single edited band was further confirmed by another round of plaque purification to obtain stocks with 100% of the plaques showing only one single edited band. Sequence analysis of the edited band confirmed that it represented the potential end joining product of the DNA ends from cleavage at the predicated Cas9 target sites (Figure 2b). Interestingly, the sequences of all three plaques being analyzed from the first round of plaque purification are the same. The existence of more variation of the edited sequence requires further screening. These experiments demonstrated that the transfection of the Cas9/gRNA construct carrying two gRNAs can be used to delete defined pieces of the viral genome efficiently.

### 3.2. Construction of Meq Deletion Mutant CVI988-ΔMeq Using CRISPR/Cas9 System

Having demonstrated that the *pp38* gene can be deleted using the CRISPR/Cas9 system with 12.5% efficiency (1/8, edited/unedited plaques) from first round screening; next we used four pairs of gRNAs targeting the *Meq* gene in an attempt to measure the knockout efficiency between the different gRNAs by using one gRNA at N-terminus combined with each of the four different gRNAs at C-terminus of the *Meq* gene. Four pairs of gRNAs were cloned into pX330A-1x2, generating Cas9/gRNA expression vectors Meq-gNC1, Meq-gNC2, Meq-gNC3 and Meq-gNC4. Using the transfection/infection/plaque purification strategy described above, 16 plaques of each Meq-gNC1 and Meq-gNC2 editing and 8 plaques of each Meq-gNC3 and Meq-gNC4 editing were screened by PCR, using primers at both ends of the *Meq* coding sequence. The expected size of the PCR products should be 1185 bp for unedited viruses and ~111 bp, ~171 bp, ~95 bp and ~166 bp for gRNAs gNC1, gNC2, gNC3 and gNC4 edited viruses, respectively. As shown in Figure 2c, the editing efficiency varied between the different pairs of gRNAs, with 31.25% (5/16) for gNC1, 56.25% (9/16) for gNC2, 75% (6/8) for gNC3 and 25% (2/8) for gNC4, respectively. Most of the plaques containing the edited bands also contained the unedited band, indicating the mixed population. More non-specific bands were observed for the last 10 lanes due to the larger amount of DNA being loaded. One potential edited band from each pair of gRNAs editing was sequenced. As expected, the edited bands corresponded to the ligation of the DNA ends that resulted from cleavage of two predicated target sites (Figure 2d). One of the edited plaques was further purified and used for subsequent characterization.

### 3.3. Characterization of Mutant CVI988 Viruses

**Figure 3:**
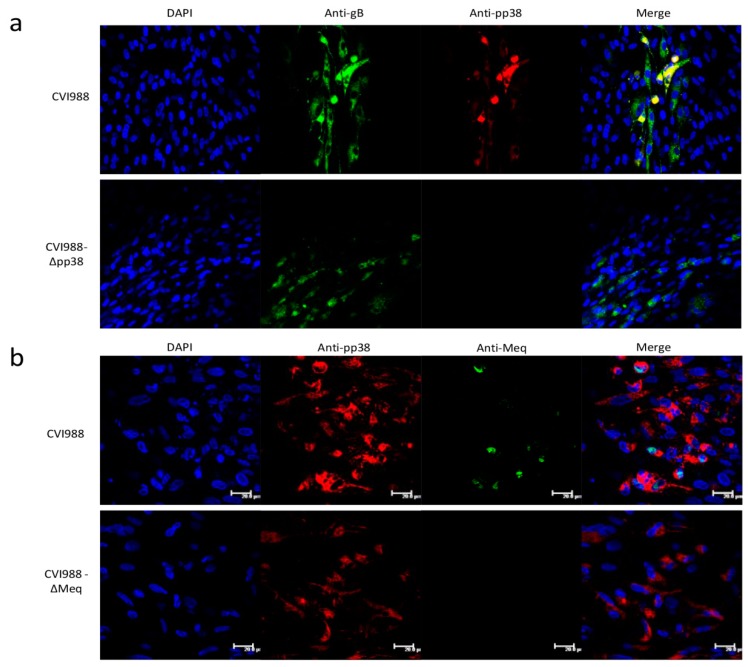
Confirmation of the mutant viruses by IFA. (**a**) Confirmation of *pp38 gene deletion* in infected CEF by IFA with anti-*pp38* monoclonal antibody BD1 (red), anti-gB monoclonal antibody (green) staining was used as infection control. (**b**) Confirmation of *Meq* gene deletion in infected CEF by IFA with anti-*Meq* monoclonal antibody FD7 (green), anti-*pp38* monoclonal antibody BD1 staining (red) was used as a control (scale bar = 20 μm).

To confirm the deletion of the *pp38* gene, virus-infected CEFs were examined by IFA with monoclonal antibodies BD1 (pp38-specific) and HB3 (gB-specific). As expected, both CVI988 and CVI988-Δ*pp38* expressed gB, whereas only CVI988 expressed *pp38* but not CVI988-Δ*pp38* (Figure 3a). The deletion of *Meq* was assessed by IFA with the monoclonal antibody FD7 (anti-Meq) and BD1 (anti-pp38). As expected, CVI988 virus expressed both *pp38* and *Meq*, whereas CVI988-Δ*Meq* expressed *pp38* but not *Meq* (Figure 3b). These results showing the lack of expression of the specific viral proteins further confirmed the successful deletion of *pp38* and *Meq* in CVI988.

**Figure 4:**
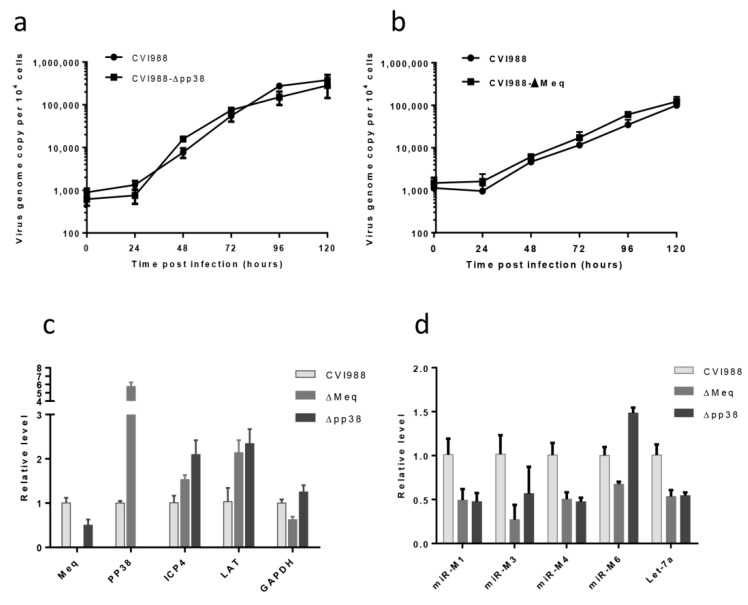
Characterization of mutant viruses. In vitro growth rates of CVI988-Δ*pp38* (**a**) and CVI988-Δ*Meq* (**b**) along with the parental CVI988 measured from the viral genome copy numbers, determined using TaqMan real-time qPCR on DNA extracted from CEF, harvested at various time points after inoculation. Viral genome copy numbers per 10,000 cells (shown with 95% confidence intervals) are shown on the y axis. There was no difference in the replication of parental CVI988 and the mutant viruses. (**c**) Relative expression of MDV genes *Meq*, *pp38*, *ICP4*, *LAT* and host gene *GAPDH* measured by qRT-PCR (normalized to MDV gene *gB*) in RNA extracted from CEF infected with the parental CVI988 and the different mutant viruses. Results represent the mean of triplicate assays with error bars showing the standard errors of the mean. (**d**) Relative expression of MDV-1 miRNAs miR-M1, miR-M3, miR-M4, miR-M6 and host miRNA *let-7a* measured by qRT-PCR (normalized to MDV1-miR-M7) in RNA extracted from CEF infected with the parental CVI988 and the different mutant viruses. Results represent the mean of triplicate assays with error bars showing the standard errors of the mean.

Next we wanted to examine the biological effects of the deletion of these viral genes on the viruses. For this, we determined whether the growth curves of the mutant viruses were comparable to that of the parental CVI988 virus. For this, primary CEFs in 6-well plates were infected with 100 pfu of either the parental CVI988 or CVI988-Δ*pp38*/CVI988-Δ*Meq*. At 24 h intervals for 6 days, DNA was extracted from the infected cells and virus replication rates were measured by qPCR that determines the viral genome copy numbers per 10,000 cells. The qPCR data on various days post-infection did not show significant differences between the parental viruses and the mutant virus for both CVI988-Δ*pp38* (Figure 4a) and CVI988-Δ*Meq* (Figure 4b).

To analyze the effect of *pp38* and *Meq* deletion mutants on MDV gene expression, we compared the relative levels of transcripts of selected MDV genes *Meq*, *pp38*, *ICP4* and *LAT*, by quantitative RT-PCR in CEF infected with wild type CVI988 or the mutant viruses. Host gene GAPDH was included as a control. As expected, the absence of the *pp38* transcript in the CVI988-Δ*pp38* virus infected cells and *Meq* transcript in the cells infected by CVI988-Δ*Meq* virus, further confirmed the successful deletion of these genes in the corresponding mutant viruses. As demonstrated in Figure 4c, the expression of the other selected genes was not affected by deletion of *Meq* or *pp38*. We also examined the expression of MDV-encoded miRNAs miR-M1, miR-M3, miR-M4, miR-M6 and miR-M7 on RNA extracted from CEF infected with wild type CVI988 and the mutant viruses. The expression of selected miRNAs was normalized to the viral miRNA MDV1-miR-M7 which is from the distal end of the deleted genes. q-RT-PCR data of these miRNAs indicated that the deletion of the *Meq* or *pp38* gene did not affect the expression of MDV-encoded miRNAs (Figure 4d).

## 4. Discussion

CRISPR/Cas9 gene editing has recently been employed to manipulate the genomes of a number of large DNA viruses. Following the successful application of the CRISPR/Cas9 system on HVT genome editing for gene disruption [38] and the knock-in of specific sequences [39] where the indel mutations at a Cas9 cleavage site in HVT genome are generated by a single gRNA at a high frequency, we report here the first use of this technology to introduce targeted mutations into the MDV-1 genome. We show that the NHEJ repair pathway can also be used to generate defined gene knockout viruses by eliminating the sequence between two Cas9 cleavage sites. To our knowledge, this is the first study to demonstrate effective use of the CRISPR/Cas9 system in viral gene knockout in MDV-1 infected CEF for mutant virus generation, in studying the viral gene functions.

Using a simple transfection/infection methodology in CEF cells, we demonstrate that CRISPR/Cas9 editing can efficiently generate targeted gene knockout mutants with two major MDV-1-encoded genes, *pp38* and *Meq*. We utilized a dual gRNA expression vector px330A-1x2 expressing two gRNAs targeting both ends of the gene of interest to ensure the simultaneous cleavage of the two target sites. Due to the cell associated feature of MDV, first round plaques are often the mixture of unedited and edited viruses. This is evidenced by the amplification of two PCR products with the primer pair outside of the target sites; the longer product representing the unedited sequence and the shorter product representing the edited sequence. For *pp38* deletion, the positive rate is 12.5% with one pair of gRNAs being tried. The pure population of a virus can be obtained with one more round of plaque purification. This has been evidenced from the following round of plaque purification, which showed all of the plaques contained only the edited band. Consistent with a previous study [14], in vitro growth characteristics of the *pp38* deleted virus was similar to the parental virus, showing that *pp38* is not essential for virus replication in cultured cells.

For the *Meq* deletion, we compared the knockout efficiency by using one gRNA at the N-terminus of *Meq* combined with one of the four gRNAs at the C-terminus. Indeed, the results show that editing rates differ between different gRNAs, ranging from 25% to 75%. This indicated that it is necessary to test more than one pair of gRNAs for achieving the most efficient cleavage.

Following the development of BAC containing the full length genomes of MDV-1 for efficient manipulation of the MDV-1 genomes to study gene functions, significant progress has been made in the last decade for direct examination of the role of a number of viral genes in pathogenesis in infection models in natural chicken hosts. We demonstrated here that CRISPR/Cas9 mutagenesis is a technically simple alternative to BAC mutagenesis that eliminates the need for a BAC intermediate. This methodology could be used to genetically manipulate low-passage clinical isolates. As shown in *pp38* deletion, a working stock of mutant viruses can be generated by CRISPR/Cas9 editing in as few as two passages. It can also overcome the limitation of reduced replication observed by BAC viruses and the introduction of mutations during the repeated virus passages for the generation of a BAC clone.

In summary, we have successfully deleted *pp38* and *Meq* genes of vaccine strain CVI988 of MDV using the CRISPR/Cas9 system. Both mutant viruses show similar growth kinetics with the parental viruses. Although only two genes have been knocked out, it will be feasible to try more genes using the same approach for gene function studies. As of other mutagenesis strategies for viral gene deletion, the inducible system has to be in place to overcome the limitation when the targeted gene is essential for in vitro replication, such as glycoprotein B. The off target effect has been demonstrated in a number of CRISPR/Cas9 editing studies. The off target mutation is not investigated here; whole viral genome sequencing is required for this purpose when the functional study is carried out. The same approach can also be extended to different pathotypes of MDVs and in MDV-1-transformed cell lines to investigate the role of viral genes in tumor cells. Gene knockout mutagenesis of the MDV strain described here provides the opportunity to exploit the power of CRISPR/Cas9 gene editing to identify molecular determinants of MDV oncogenicity.

## Figures and Tables

**Table 1:** List of primer sequences for gRNA cloning and identification of mutants.

Primer	Sequence (5′-3′)
pp38-gN-F	CACCGGGTATGTTAGTCGGTAGAA
pp38-gN-R	AAACTTCTACCGACTAACATACCC
pp38-gC-F	CACCGCTCGTCGGCGACCCCTGCG
pp38-gC-R	AAACCGCAGGGGTCGCCGACGAGC
Meq-gN-F	CACCGCGACCCGAGAGAAAGATCG
Meq-gN-R	AAACCGATCTTTCTCTCGGGTCGC
Meq-gC1-F	CACCGCCGTAGACTGAGTATCCGA
Meq-gC1-R	AAACTCGGATACTCAGTCTACGGC
Meq-gC2-F	CACCGCTTTATGCTCGTCTTACCG
Meq-gC2-R	AAACCGGTAAGACGAGCATAAAGC
Meq-gC3-F	CACCGTACTCAGTCTACGGTCTGG
Meq-gC3-R	AAACCCAGACCGTAGACTGAGTAC
Meq-gC4-F	CACCGGAATCCTGTTCGGGATCCT
Meq-gC4-R	AAACAGGATCCCGAACAGGATTCC
pp38-N-F	GATTCCACCTCCCCAGAATCC
pp38-C-R	TTCGAAGCAGAACACGAAGGG
Meq-N-F	ATGTCTCAGGAGCCAGAG
Meq-C-R	TCAGGGTCTCCCGTCA
